# Spatio-Temporal Variation and Prediction of Ischemic Heart Disease Hospitalizations in Shenzhen, China

**DOI:** 10.3390/ijerph110504799

**Published:** 2014-05-06

**Authors:** Yanxia Wang, Qingyun Du, Fu Ren, Shi Liang, De-nan Lin, Qin Tian, Yan Chen, Jia-jia Li

**Affiliations:** 1 School of Resource and Environmental Science, Wuhan University, 129 Luoyu Road, Wuhan 430079, China; E-Mails: wangwangyanxia@163.com (Y.W.); qydu@whu.edu.cn (Q.D.); tianqin@whu.edu.cn (Q.T.); 2 Key Laboratory of GIS, Ministry of Education, Wuhan University, 129 Luoyu Road, Wuhan 430079, China; 3 Shenzhen Center for Health Information, Renmin Road North 2210, Luohu District, Shenzhen 518001, China; E-Mails: ldn308@163.com (D.L.); chy@newhealth.com.cn (Y.C.); jiajiali831@gmail.com (J.L.)

**Keywords:** ischemic heart disease (IHD), spatio-temporal analysis, spatial disparities, grey model, China

## Abstract

Ischemic heart disease (IHD) is a leading cause of death worldwide. Urban public health and medical management in Shenzhen, an international city in the developing country of China, is challenged by an increasing burden of IHD. This study analyzed the spatio-temporal variation of IHD hospital admissions from 2003 to 2012 utilizing spatial statistics, spatial analysis, and space-time scan statistics. The spatial statistics and spatial analysis measured the incidence rate (hospital admissions per 1,000 residents) and the standardized rate (the observed cases standardized by the expected cases) of IHD at the district level to determine the spatio-temporal distribution and identify patterns of change. The space-time scan statistics was used to identify spatio-temporal clusters of IHD hospital admissions at the district level. The other objective of this study was to forecast the IHD hospital admissions over the next three years (2013–2015) to predict the IHD incidence rates and the varying burdens of IHD-related medical services among the districts in Shenzhen. The results show that the highest hospital admissions, incidence rates, and standardized rates of IHD are in Futian. From 2003 to 2012, the IHD hospital admissions exhibited similar mean centers and directional distributions, with a slight increase in admissions toward the north in accordance with the movement of the total population. The incidence rates of IHD exhibited a gradual increase from 2003 to 2012 for all districts in Shenzhen, which may be the result of the rapid development of the economy and the increasing traffic pollution. In addition, some neighboring areas exhibited similar temporal change patterns, which were also detected by the spatio-temporal cluster analysis. Futian and Dapeng would have the highest and the lowest hospital admissions, respectively, although these districts have the highest incidence rates among all of the districts from 2013 to 2015 based on the prediction using the GM (1,1). In addition, the combined analysis of the prediction of IHD hospital admissions and the general hospital distributions shows that Pingshan and Longgang might experience the most serious burden of IHD hospital services in the near future, although Futian would still have the greatest number and the highest incidence rate of hospital admissions for IHD.

## 1. Introduction

Ischemic heart disease (IHD), also known as coronary heart disease (CHD) [1], is caused by the buildup of plaque along the inner walls of the coronary arteries, which narrows the arteries and reduces blood flow to the heart; IHD is the leading cause of death worldwide [2,3,4]. In 2004, the number of IHD-related deaths was 7.2 million, accounting for 12.2% of all deaths and 5.8% of all years of life lost, and 23.2 million people experienced moderate or severe disability due to IHD [5]. As the most common type of heart disease, the projected total costs of IHD will increase from 46.8 billion dollars in 2015 to 106.4 billion dollars in 2030, an increase of 123.75%, as estimated by the American Heart Association [6]. In China, heart disease has also been a leading cause of death in the past two decades. Among the 15 major diseases among urban residents, mortality from heart disease was ranked 3rd, 4th, 3rd, 3rd, and 2nd in 1990, 1995, 2000, 2005, and 2010, respectively [7]. In particular, mortality from IHD is approximately 95.97 per 100,000 people in China in 2011, accounting for approximately 72.64% of the total deaths from heart disease [7]. Furthermore, the annual direct costs of cardiovascular disease are approximately 4% of the gross national income in China [8], and IHD is a chief component of cardiovascular diseases. Therefore, it is necessary to analyze the spatio-temporal variation of IHD hospitalizations using spatial statistics and spatial analysis and to forecast the hospital admissions and incidence rate related to IHD and the corresponding burden on medical services using the grey model GM (1,1). Such analyses will facilitate the development of appropriate, region-specific measures for the prevention and management of IHD.

Because IHD is the leading cause of death, many studies have investigated the distribution of IHD and attempted to forecast its future distribution. The traditional distribution studies can be classified into two types: those investigating time trends and those investigating health disparities. The studies investigating time trends analyze the changes in one or more countries over time (usually with the year as the unit) [9,10,11,12]. The health disparity studies are mainly focused on the diversity among different social, racial, or ethnic populations [8,13,14,15,16,17,18]. With the development of Geographic Information System (GIS) technology and its applications in epidemiology [19], heath disparity studies have been expanded to include spatial dimensions using spatial analysis, spatial statistics, and mapping visualizations [20,21], such as geographic distribution [22,23] and the spatial heterogeneity of health issues related to environmental inequalities (such as air pollution) [24,25,26]. Unfortunately, the above distribution studies are generally analyzed according to traditional statistical methods and are based on large areas. Furthermore, there is limited research on the spatial variation of IHD hospitalizations, and the factors affecting this variation, such as socioeconomic status and environmental conditions, could vary among different regions. Prediction studies usually use models to forecast mortality or incidence. For example, Wilson *et al.* [27] used risk factor categories (age, diabetes, smoking, JNC-V blood pressure categories, NCEP total cholesterol, and LDL cholesterol categories) to predict gender-specific CHD risk. Murray and Lopez [3], as well as Mathers and Loncar [2], utilized three types of projection models (baseline, pessimistic, and optimistic) for both sexes and seven age groups to predict mortality rates for the cause-related clusters based on the four independent variables of income per person, average years of schooling per adult, smoking intensity, and time. In addition to the multiple-factor prediction model, time series models, such as the Markov computer simulation, have been used to predict morbidity, mortality, or costs [28]. However, such multiple-factor prediction models must be based on comprehensive and accurate data regarding the underlying causes of the diseases and the related factors, which could be difficult to obtain, and assumptions must be made for the other models. Therefore, predicting the future disease incidence or mortality using a grey model based directly on previous observations seems to be a practical approach.

Since it was introduced in 1982 by Deng, the grey system theory has become very popular due to its ability to address systems that have partially unknown parameters [29,30]. In addition, grey models (GMs) require only a limited amount of data (at least four time series data points) to forecast the development of the unknown systems [31]. Due to its advantages, the GM has been successfully applied to many disciplines, including economics, sociology, engineering, and others, and has demonstrated satisfactory results in recent years [32,33]. In particular, the model has been applied in the predictions of some similar aspects of IHD hospitalizations. Li *et al.* [34] applied the GM and the grey relational analysis to forecast the development of six indicators of the financial burden of patients between 2012 and 2015. Wu and Chen [32] used the grey model GMC (1,n) combined with a grey relational analysis to predict the population that would have access to the internet based on 12 years of observed data. Mao and Chirwa [35] applied grey model GM(1,1) to estimate vehicle fatality risk on the basis of the observed data from 1966 to 2001 in the USA and from 1969 to 2000 in the UK.

In addition, as fiscal and economic administrations have gradually become more decentralized during the Reform and Opening period in China, urban governments have enjoyed more autonomy in resource allocation, urban planning, and economic policy [36]. Moreover, the incidence rate of IHD hospitalizations can be reduced by high-quality primary care [37,38,39]. Therefore, a better understanding of the spatio-temporal characteristics and the prediction of IHD within a city would facilitate the identification of areas and populations at high risk. Such analyses would enable decision makers to formulate appropriate urban public health policies and efficiently allocate public health resources for the prevention and treatment of IHD [40]. However, previous studies have mainly focused on the global, national, or regional levels [41].

Shenzhen is considered to be one of the fastest-growing cities in the World; it has developed into an international city from a small fishing village approximately 30 years ago [42,43]. The increasing burden of chronic diseases, such as IHD, accompanied by the rapid expansion of urbanization, is a major challenge to Shenzhen health and medical management [44,45]. Furthermore, the population grew to 10.5 million in 2012 from 0.3 million in 1979; the resulting almost 35-fold increase in the population [46,47] was almost entirely a result of migration, with an average age of 30 years [48]. The large migrant population makes it difficult for the local health authorities to control and prevent the transmission of infectious diseases (such as HIV/AIDS), chronic diseases (such as IHD and hypertension), and psychological disorders (such as depression) [49]. Moreover, there are substantial variations in the association between health and migration, and there are health disparities between the local residents and the migrant population and health disparities within the migrant population [50,51]. Therefore, lifestyle health promotion, disease surveillance, and disease prevention are important aspects of the healthcare system [44]. The total annual investment in medicine and healthcare increased to 7.87 billion Yuan in 2011 from 1.30 billion Yuan in 2003 [46,52]. In addition, the mortality from heart disease has always been ranked highly among the major causes of death (e.g., ranked 3rd in 2003 and 2nd in 2012), although the mortality has shown a downward trend (*i.e.*, the mortality was decreased to 0.82 in 2012 from 1.27 in 2003) [53,54]. As a result, it is important to analyze the spatio-temporal variations and to predict IHD hospitalizations in Shenzhen for effective public health surveillance and management and the efficient allocation of healthcare resources to develop public healthcare and promote equal health services.

The present study aimed to (a) clarify the spatial and temporal characteristics of IHD hospitalizations at the district level in Shenzhen, China from 2003 to 2012 utilizing spatial statistics and analyses and (b) predict the IHD hospital admissions and incidence rates based on the GM (1,1) over the next three years to illustrate the differential burden of IHD hospital services in the districts. The study investigated: (1) the temporal trends of IHD from 2003 to 2012, (2) the geographical distribution of IHD within Shenzhen at the district level, (3) the spatio-temporal clusters and variations of IHD, and (4) the prediction of IHD-related hospital admissions and incidence rates from 2013 to 2015 and the concomitant district-level disparities in the hospital service burdens.

## 2. Materials and Methods

### 2.1. Study Area

**Figure 1:**
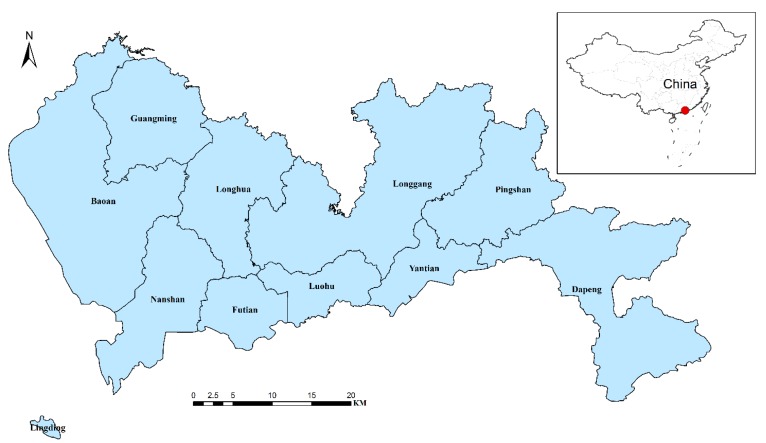
A map of Shenzhen and its location in China.

Shenzhen is the oldest and most successful Special Economic Zone (designated in 1979) in China. The city is located in the southern portion of southern China’s Guangdong province and is immediately north of Hong Kong (Figure 1). There are six administration districts and four administration units in Shenzhen: Futian, Luohu, Nanshan, Yantian, Longgang, Baoan, Guangming, Pingshan, Longhua, and Dapeng. The Guangming and Longhua units were created from the Baoan district in 2007 and 2011, respectively; the Pingshan and Dapeng units were created from the Longgang district in 2009 and 2011, respectively. In this study, the study area includes the ten districts under the administration of Shenzhen according the latest administrative divisions.

### 2.2. Data Description

#### 2.2.1. IHD Data

The IHD data for hospitalized patients from 2003 to 2012 were obtained from the Shenzhen Center for Health Information. These data included age, gender, address, diagnosis, and other information. The diagnoses were recorded based on the 10th revision of the International Statistical Classification of Diseases and Related Health Problems (ICD-10), which coded for diseases, signs and symptoms, abnormal findings, complaints, social circumstances, and external causes of injury or disease [55]. Subjects were classified as having IHD (ICD-10 I20-I25), angina pectoris (ICD-10 I20), other acute ischemic heart diseases (ICD-10 I24), chronic ischemic heart disease (ICD-10 I25), acute myocardial infarction (ICD-10 I21), certain current complications following acute myocardial infarction (ICD-10 I23), or subsequent myocardial infarction (ICD-10 I22).

#### 2.2.2. Population Data

The annual population data for each administrative district from 2003 to 2011 were mainly obtained from the Shenzhen Statistical Yearbooks from 2004 to 2012 [46,52,56,57,58,59,60,61,62]. Due to the changes in the administrative divisions in 2007, 2009, and 2011, the population data for Guangming (2004–2006), Pingshan (2004–2007), Longhua (2004–2011), and Dapeng (2004–2011) were not included in the Shenzhen Statistical Yearbooks. Therefore, the Baoan and Longgang Statistical Yearbooks from 2004 to 2011 [63,64,65,66,67,68,69,70,71,72,73,74,75,76,77,78] were used to provide the missing data. In addition, the population data for each district in 2012 were extracted from the Statistical Communiqué of Shenzhen in the 2012 National Economic and Social Development Report [47].

#### 2.2.3. Spatial District Data and General Hospital Data

**Figure 2:**
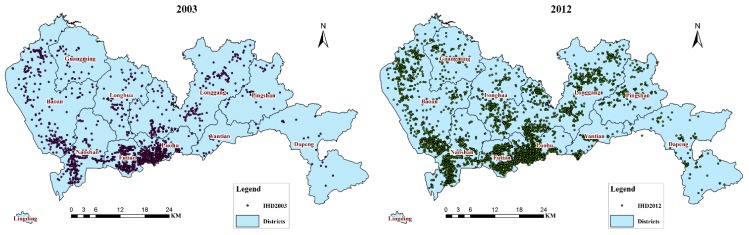
Distribution map of annual IHD hospitalizations, with 2003 and 2012 as examples.

To investigate the locations of IHD, spatial data sets on the administrative units and roads are required for address geocoding [79]. Using these data, the IHD attribute data were converted into spatial data (Figure 2). In addition, a spatial statistical analysis of the IHD cases in each district was conducted based on the administrative spatial data.

**Figure 3:**
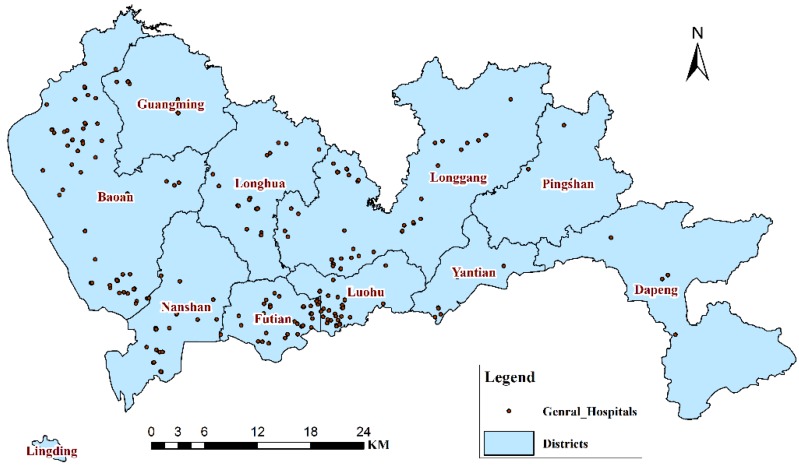
Distribution map of general hospitals in Shenzhen, 2011.

The locations of the general hospitals in 2011 (Figure 3) were obtained to analyze the potentially unequal burdens on medical service resources based on the predictions of IHD hospital admissions from 2013 to 2015.

### 2.3. Methodology

#### 2.3.1. Incidence Rate and Standardized Ratio Calculation

The incidence rate (IR) and the standardized ratio (SR) were used to represent disease risk across Shenzhen in this study [38,45,80,81] to identify districts with higher or lower disease risks and to capture the temporal and spatial variations. The IR is expressed as hospital admissions per 1,000 residents using the total population of the corresponding district as the standard, which can be described as follows:

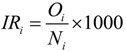
(1)
where *Q_i_* and *N_i_* are the hospital admissions for IHD and the total population in the *i*th district, respectively.

The SR is expressed as a ratio of the number of observed cases to the number of expected cases in the total population of the corresponding district, which can be expressed as follows:

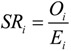
(2)
where *Q_i_* and *E_i_* represent the observed and expected numbers of IHD hospital admissions in the *i*th district, respectively. In addition, *E_i_* is calculated by multiplying the general IR of Shenzhen by the population of the *i*th district and can be expressed as follows:

*E_i_* = *G_IR_* × *N_i_* (3)

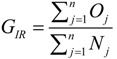
(4)
where *G_IR_* is the general IR of Shenzhen, which can be calculated by Equation (4). In addition, *n* is the number of districts administered by Shenzhen and *Q_j_* and *N_j_* are the number of observed cases and the population in the *j*th (*j* = 1,2,…*n*) district, respectively.

#### 2.3.2. Spatio-temporal Variation Analysis

A GIS-based global spatio-temporal map was applied to the IHD IRs and SRs to analyze their spatio-temporal distribution throughout Shenzhen. The IRs of IHD were analyzed at yearly intervals separately for each district to exhibit the temporal distribution and patterns using ArcGIS 10.1 to map the annual IRs from 2003 to 2012 for each district. Furthermore, the average IRs related to IHD from 2003 to 2012 for all of the districts were classified into four ranks according to the standard deviation: (1) the first interval was from 0.350 to 0.710 and represented the standard deviations from the mean value of all of the average IRs smaller than −0.29; (2) the second interval was from 0.711 to 1.180 and represented the standard deviations from the mean value of all of the average IRs between −0.29 and 0.61; (3) the third interval was from 1.181 to 1.650 and represented the standard deviations between 0.61 and 1.5; and (4) the fourth interval was from 1.651 to 2.070 and represented the standard deviation from the mean average IRs larger than 1.5. The SRs of IHD were analyzed at the district level separately for each year to indicate the relative risk of IHD in the districts, *i.e.*, the annual spatial distribution of IHD SRs. The SRs were classified into five ranks based on the K-mean clustering method, *i.e.*, the first rank of SRs, from 0 to 0.580, represented the lowest relative risk among the districts; the second rank of SRs, from 0.581 to 0.890, represented a low relative risk; the third rank of SRs, from 0.891 to 1.270, represented a middle relative risk; the fourth rank of SRs, from 1.271 to 1.840, represented a high relative risk; and the fifth rank of SRs, from 1.841 to 2.500, represented the highest relative risk. Furthermore, the changes in the SRs of IHD were analyzed for each district every three years and during the entire study period to identify the changing patterns of the SR rank.

There are many spatial statistical tools to measure the central tendency and the dispersion of point events to describe the characteristics of a univariate distribution, including the mean center (MC), the standard deviational ellipse (SDE), and the standard distance [82,83,84,85]. The MC and the SDE tools in ArcGIS 10.1 were analyzed to identify the spatio-temporal changes of IHD hospital admissions in Shenzhen from 2003 to 2012. The MC identifies the geographic center of a set of points to measure the central tendency, which is calculated as follows:

*MC_t_* = (*X_t_*, *Y_t_*) (5)

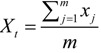
(6)

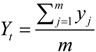
(7)
where *MC_t_* denotes the coordinates of the MC in the *t*th year, *m* is the number of points over the study area in the *t*th year, and *x_j_* and *y_j_* are the coordinates of the *j*th (*j* = 1,2,…*m*) point in the *t*th year. The IHD MCs of the same geographic area in a time series could reveal the movement of the IHD central tendency. The MCs of the address locations of the IHD hospital admissions from 2003 to 2012 were measured to indicate the yearly movement of the IHD central tendency. In addition, the MCs of annual population from 2003 to 2012 were measure to compare with the change of annual MCs of IHD.

The SDE measures the spatial distribution of points around their MCs to describe the dispersion and orientation. The SDE method was first proposed by *Furfey* [86], and its computational procedure was provided by *Ebdon* [83]. The SDE parameter could be calculated as follows:

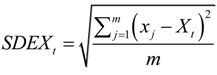
(8)

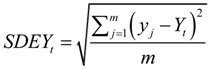
(9)

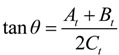
(10)

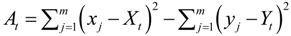
(11)

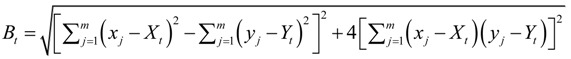
(12)

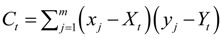
(13)
where *SDEX_t_* and *SDEY_t_* represent the two standard distances (long and short axes, respectively) of the standard deviational ellipses in the *t*th year, *X_t_* and *Y_t_* are the coordinates of the MC in the *t*th year calculated by Equations (5–7), *m* is the number of points over the study area in the *t*th year, and are the coordinates of the *j*th (*j* = 1,2,…*m*) point in the *t*th year, and *θ_t_* is the rotation angle of the standard deviational ellipses in the *t*th year. The SDE was used to describe the spatial distribution of IHD hospital admissions. A SDE could be derived for annual IHD hospital admissions, and multiple SDEs of IHD hospital admissions could be compared against each other to reveal the extent of spatial correlation among the annual IHD hospital admissions. That is, the area of overlap among these SDEs indicated the degree of spatial correlation among the annual IHD hospital admissions, while the areas where the annual SDEs did not overlap represented spatial segregation [87].

SatScan, which uses the Kulldorff method of retrospective space-time scan statistics based on a discrete Poisson model, was used to detect IHD clusters in individual districts from 2003 to 2012. The space-time scan statistics is defined by a cylindrical window with a circular geographic base and a height corresponding to time [88,89]. The SatScan can detect several cluster centroids located throughout the study area, which are surrounded by other points with the radius of the base varying continuously according to the population range of the area, from zero to the maximum cluster size of the total population that might be at risk. In this study, the default maximum spatial cluster size of 50% was selected for the space-time cluster analysis. Furthermore, the log likelihood ratio (LLR) was used to calculate the difference of the incidence inside and outside the windows:

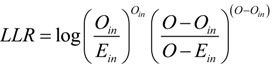
(14)
where *O_in_* and *E_in_* denote the numbers of actual and expected cases in the window, respectively. *E_in_* can be calculated by Equations (3) and (4). The most likely cluster is the scan window with the largest LLR value, and the secondary clusters are the other scan windows with statistically significant LLR values. The IHD hospital admissions and population of each district in each year and the coordinates of each district were included to obtain the most likely cluster in which the districts and time frame have the largest LLR and the maximum relative risk.

#### 2.3.3. Prediction Analysis

The grey model GM (1,1) was used to predict the development trend of IHD IRs and the corresponding medical burden in the near future in Shenzhen based on the existing data. The GM (1,1) is a time series prediction mode that consists of three basic operations: accumulated generation, inverse accumulated generation, and grey modeling [35,90]. The process of GM (1,1) for forecasting is constructed as follows:

Step 1: Assume an initial time sequence consisting of n + 1 values:

*X* = {*x*_0_,*x*_1_,…,*x_t_*,…,*x_n_*} (15)

where *x_t_* is the value at time *t*(*t* = 0,1,2,…,*n*) and *n* must not be smaller than three.

Step 2: Establish a new time sequence *Y* based on the initial sequence *X* through the accumulated generation operation to reduce the inconsistencies of building a GM and to weaken the variation tendency. *Y* can be calculated as follows:

*Y* = {*y*_0_,*y*_1_,…,*y_t_*,…,*y_n_*} (16)

where *t* = 0,1,2,…,*n*, and:

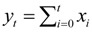
(17)

Moreover, a mean time sequence *Z* is calculated based on the accumulated sequence *Y*, *i.e.*:

*Z* = {*z*_1_,*z*_2_,…,*z_t_*,…,*z_n_*} (18)

where *t* = 0,1,2,…,*n*, and:

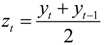
(19)

Step 3: Construct GM (1,1) based on the first-order differential equation:

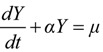
(20)

and the difference equation:

*X* + *αZ* = *μ* (21)

The solution of Equation (20) could be obtained as follows:

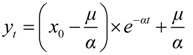
(22)
where *t* = 0,1,2,…,*n*, and:

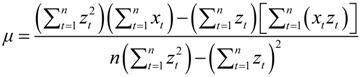
(23)

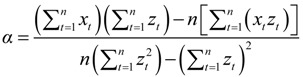
(24)

Step 4 Evaluate the predicted value of *x_t_* at time *t*:

*x_t_* = *y_t_* - *y_t-1_* (*t* = 0,1,2,…,*n*) (25)

In addition, the posterior deviation ratio *c* and the small error probability *p* are calculated to estimate this prediction as follows:

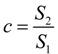
(26)

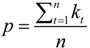
(27)
where:

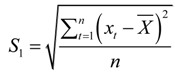
(28)

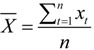
(29)

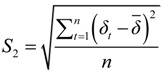
(30)

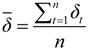
(31)

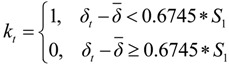
(32)

*δ_t_* = *x_t_* - *x_t_* (*t* = 0,1,2,…,*n*) (33)

The forecasting results can be classified into four types according to the values of *c* and *p*: (a) if *p*>0.95 and *c*<0.35 , then the results are “excellent”; (b) if *p*>0.80 and *c*<0.50 , then the results are “good”; (c) if *p*>0.70 and *c*<0.65 , then the results are “marginal”; and (d) otherwise, the results are “unreliable.”

**Figure 4:**
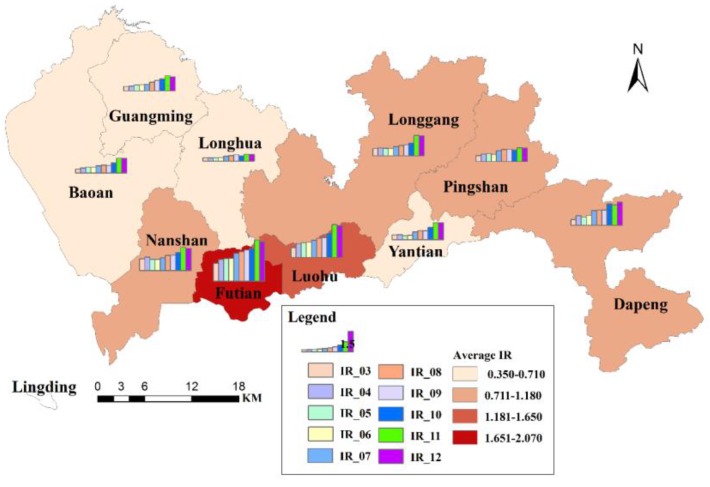
The map of the IRs and total hospital admissions of IHD from 2003 to 2012 at the district level in Shenzhen.

**Figure 5:**
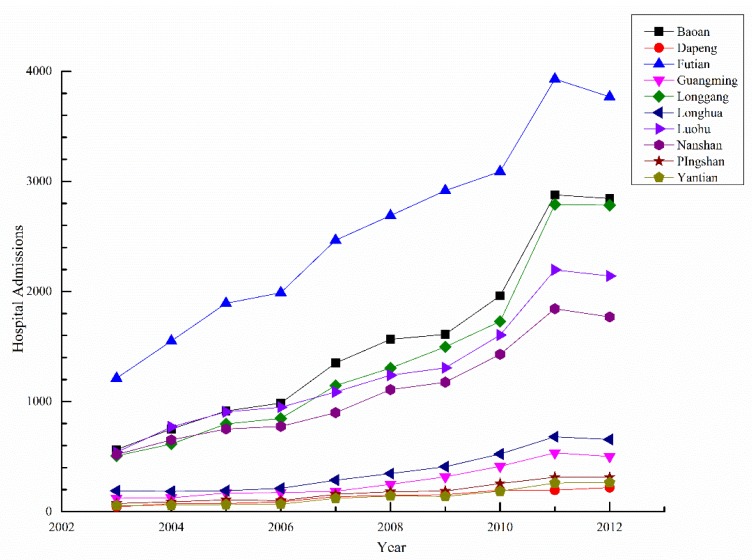
The IHD hospital admissions of each district in Shenzhen from 2003 to 2012.

First, the IHD IRs over the next three years (2013–2015) were predicted separately for each district based on the GM (1,1) using the IHD IRs from 2003 to 2012 (Figure 4) as the initial time sequence data. Second, the IHD hospital admissions over the next three years (2013–2015) were predicted separately for each district using the IHD hospital admissions from 2003 to 2012 (Figure 5) as the initial time sequence data. Finally, the IHD medical burden from 2013 to 2015 was calculated by dividing the prediction value of IHD hospital admissions by the number of hospitals (Figure 3) separately for each district.

## 3. Results and Discussion

### 3.1. Spatio-temporal Distribution

The temporal distribution of IHD hospitalizations in Shenzhen from 2003 to 2012 is shown in Figure 4. The color of each district represents the average IR related to IHD from 2003 to 2012, and the bar charts illustrate the annual IR from 2003 to 2012 (*i.e.*, IR-03 to IR-12) for each district. The largest average IR of IHD hospital admissions was in Futian; the next largest average IR of IHD hospital admissions was in Luohu, The mid-range average IRs of IHD hospital admissions were in Nanshan, Longgang, Pingshan, and Dapeng; and the smallest average IRs of IHD hospital admissions were in Baoan, Guangming, Longhua, and Yantian. The analysis reveals that the IR of each district has experienced a variable but gradually increasing trend. Most of the maxima occurred in 2011 and 2012 for all districts. It is notable that some neighboring areas exhibited similar temporal patterns. In the east (Yantian, Longgang, Pingshan, and Dapeng), the IRs from 2007 to 2009 were almost identical. In the west (Nanshan, Futian, Luohu, Baoan, Guangming, and Longhua), outbreaks occurred during the same period in 2011.

The causes of the rise of IRs for all the districts from 2003 to 2012 may be the growth of the gross domestic product (GDP) and/or the aggravation of traffic pollution and related factors, according to a previous analysis of IHD-related factors [17,26]. The GDP has grown at a rate of greater than 10% since 2003 (except for 2009), and reached a maximum of approximately 20% in 2011. The total length and area of roads increased from 2,917.8 km and 71.38 km^2^ in 2003 to 6,228 km and 90.98 km^2^ in 2011 (respective increases of 113.4% and 27.2%). In addition, the number of buses increased from 4,885 in 2003 to 14,873 in 2011 (a 204.5% increase) [46,56]. Therefore, health-related policies should be formulated to prevent overly rapid increases in the IR of IHD and its related factors in all districts. In addition, the probable causes of the IHD outbreak in 2011 in the west should be studied to provide guidelines for the prevention of IHD to identify measures for predicting future IHD outbreaks.

**Figure 6:**
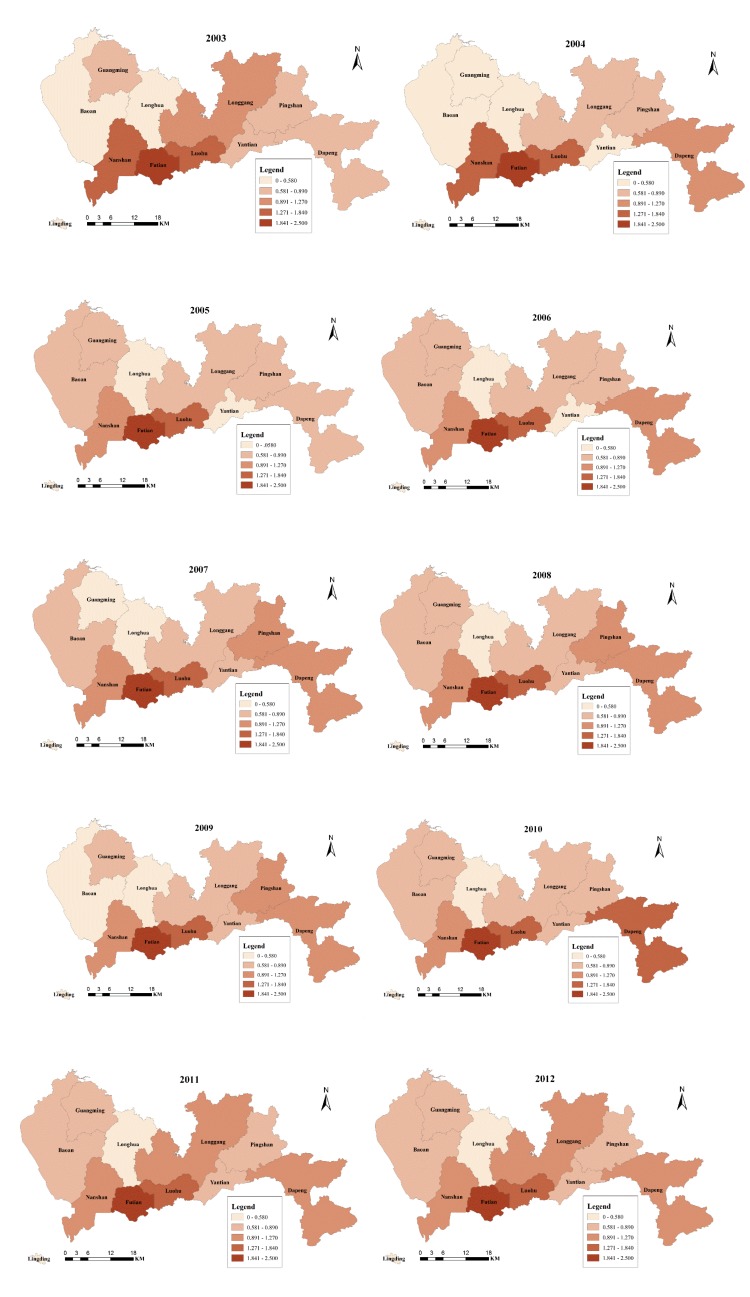
The IHD SRs of the districts in Shenzhen from 2003 to 2012.

The relative risk of IHD throughout the geographic area of Shenzhen from 2003 to 2012 is represented by the SR of each district in Figure 6. Among the districts in Shenzhen, Futian always has the highest SR, Luohu always has a high SR, and Longhua always has the lowest SR. In addition, all the districts display no changes in the SR from 2011 to 2012. As a result, Futian and Luohu should be given more attention for IHD prevention and control in the future.

**Figure 7:**
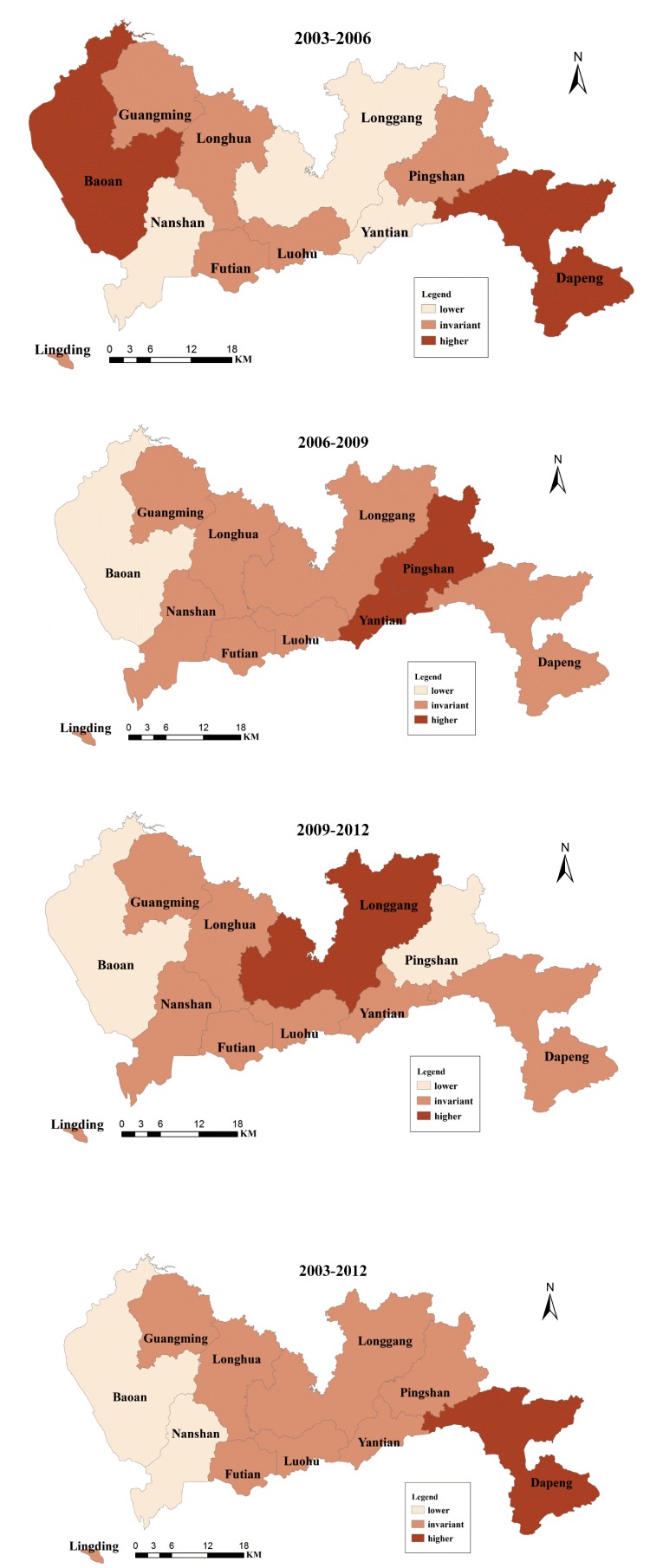
The SR changes every three years and the total SR changes for each district in Shenzhen.

The SR changes every three years and the total changes from 2003 to 2012 across Shenzhen are shown in Figure 7. The dark red color represents the SRs of the districts that experienced an increase of one rank (higher) during the time interval, the light red color represents the SRs of the districts that experienced a decrease of one rank (lower) during the time interval, and the middle red color represents the SRs of the districts that experienced no changes (invariant) during the time interval. There were no total changes in SR (2003–2012) for most of the districts, with the exceptions of Nanshan, Baoan, and Dapeng. However, these districts exhibited different change patterns during the three time intervals (2003–2006, 2006–2009, and 2009–2012). The SRs of Futian, Luohu, Guangming, and Longhua remained unchanged throughout the three time intervals. Futian and Longhua retained the same levels of SR from 2003 to 2012 (Figure 6). 

Yantian exhibited a lower SR in the first time interval, a higher SR in the second time interval, and an invariant SR in the third time interval. Longgang had a lower SR in the first time interval, an invariant SR in the second time interval, and a higher SR in the third time interval. Pingshan had an invariant SR in the first time interval and a higher SR and a lower SR in the second and the third time intervals, respectively. Nanshan had a lower SR in the first time interval and remained unchanged in the next two time intervals. Dapeng exhibited the opposite pattern of Nanshan, showing the opposite total changes from 2003 to 2012, *i.e.*, a higher SR in the first interval and no change in the next two intervals. Baoan had the same total SR change from 2003 to 2012 as Nanshan but with different patterns in the three time intervals. Baoan had a higher SR in the first time interval and lower SRs in the next two time intervals. Therefore, the spatial structures of relative risk for IHD have been consistent over the past 10 years. In particular, Nanshan and Baoan may serve as examples of successful IHD prevention for the other districts. However, Dapeng might also have taken measures to prevent further increases of IHD.

### 3.2. Spatio-temporal Clusters

**Figure 8:**
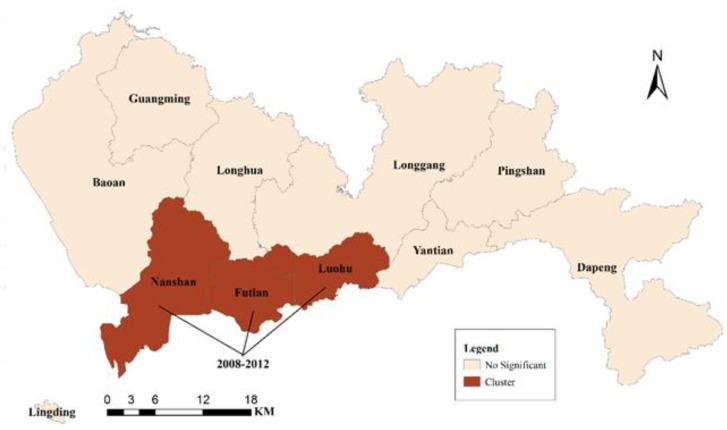
The spatio-temporal clusters of the relative risk in Shenzhen, 2003 to 2012.

The results of the cluster analysis of the IHD spatio-temporal distribution at the district level are mapped in Figure 8. The IHD exhibited a statistically significant cluster pattern in the spatio-temporal distribution, *i.e.*, Nanshan, Futian, and Luohu had the highest relative risk (2.54) between 2008 and 2012 because the *p*-value of this group was less than 0.0001 [89]. Therefore, the factors related to the cluster districts during the analyzed time period might be used to analyze the local probable causes of IHD in Shenzhen for the future prevention and management of IHD.

### 3.3. Spatio-temporal Change Analysis

**Figure 9:**
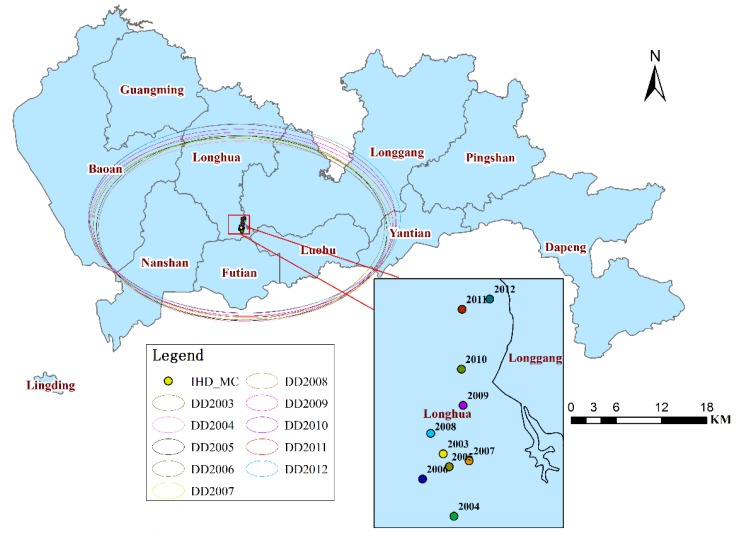
Annual mean center and directional distribution of IHD in Shenzhen from 2003 to 2012.

The investigation of the MC and the SDE analysis revealed that from 2003 to 2012, the IHD centers (Figure 9) were all located in the southeast of Longhua, near Futian and Luohu, which exhibited the highest SRs among the entire study area. 

**Figure 10:**
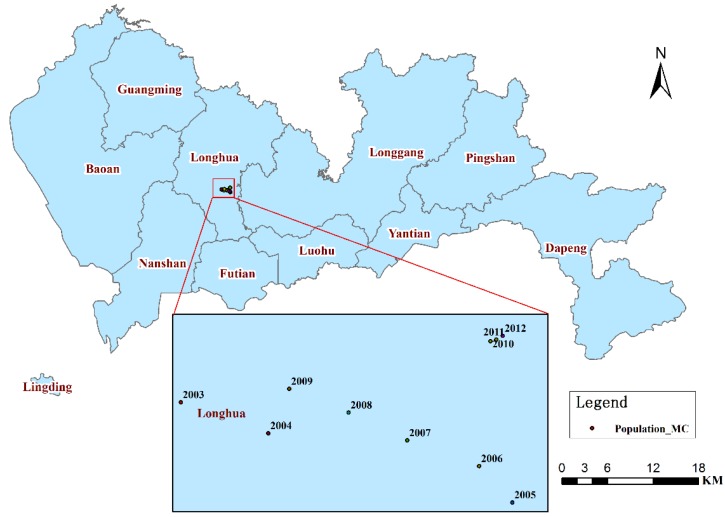
Annual mean center of the total population in Shenzhen from 2003 to 2012.

The MCs and SDEs exhibited a slight northward movement, except for in 2003 and 2006; the majority of the MCs were in the adjacent locations, and the majority of SDEs appeared in adjacent locations with similar shapes, sizes, and directions (west-east). The IHD hospitalizations have expanded to the north of Shenzhen and have a high spatial correlation of each year. In addition, the MCs of the total population exhibited a small northward movement, except for in 2003 and 2004 (Figure 10). Furthermore, he MCs of the population moved slightly northwestward from 2005 to 2009 and slightly northeastward from 2010 to 2012. Therefore, the slight northward movement of the centers of IHD hospital admissions may have been caused by the slight northward movement of the population. In particular, the MCs of the IHD hospital admissions and the total population both exhibited a slight northeastward movement from 2010 to 2012. Thus, health-related policies should be formulated to prevent this expansion or to take measures to adjust to it.

### 3.4. Predicting the Results of IHD and the Corresponding Medical Burden

The prediction results of IHD IRs and hospital admissions over the next three years are shown in Table 1 and Table 2. In general, the predictions of IHD hospital admissions are satisfactory because all of the predictions have the degree of “excellent”; however, the prediction of IHD IRs is slightly less satisfactory because there are some exceptions, which have the degree of “good”.

**Table 1:** Prediction of IHD IR based on GM (1,1).

Districts	Prediction Value			*S_1_*	*S_2_*	*c*	*p*	Degree
	2013	2014	2015					
Baoan	1.23	1.44	1.67	0.2509	0.0757	0.301714	1	excellent
Dapeng	1.96	2.22	2.51	0.3704	0.1053	0.284287	1	excellent
Futian	3.2	3.48	3.78	0.4803	0.1176	0.244847	1	excellent
Guangming	1.3	1.5	1.74	0.2585	0.059	0.22824	1	excellent
Longgang	1.66	1.95	2.28	0.3451	0.112	0.324544	1	excellent
Longhua	0.56	0.6	0.66	0.0938	0.0373	0.397655	0.8889	good
Luohu	2.65	3	3.4	0.4903	0.1273	0.259637	1	excellent
Nanshan	1.79	1.97	2.18	0.3005	0.1163	0.387022	0.8889	good
Pingshan	1.13	1.22	1.33	0.1775	0.0729	0.410704	0.8889	good
Yantian	1.58	1.91	2.3	0.341	0.0802	0.235191	1	excellent

**Table 2:** Prediction of IHD hospital admissions based on GM (1,1).

Districts	Prediction Value			*S_1_*	*S_2_*	*c*	*p*	Degree
	2013	2014	2015					
Baoan	3,532	4,197	4,988	739.5386	157.6849	0.213221	1	excellent
Dapeng	263	300	343	51.1903	12.7783	0.249623	1	excellent
Futian	4,461	4,970	5,537	773.7296	164.6242	0.212767	1	excellent
Guangming	670	807	972	144.2794	35.003	0.242606	1	excellent
Longgang	3,498	4,264	5,196	762.7598	168.6213	0.221067	1	excellent
Longhua	862	1,032	1,237	182.3981	37.1975	0.203936	1	excellent
Luohu	2,540	2,923	3,364	492.6384	112.5286	0.22842	1	excellent
Nanshan	2,157	2,477	2,843	415.7689	85.9678	0.206768	1	excellent
Pingshan	396	467	551	81.8777	17.2176	0.210284	1	excellent
Yantian	342	416	506	73.9185	16.0493	0.217122	1	excellent

As shown in Table 1 and Table 2, Longgang might surpass Baoan in IHD hospital admissions after 2014, causing Longgang to be ranked 2^nd ^among all of the districts. Futian, Luohu, and Dapeng would have the highest IHD IRs among all of the districts. In addition, Futian and Dapeng might continue to have the most and the least IHD hospital admissions, respectively; however, these districts had the highest IRs among the districts of Shenzhen during the prediction period.

**Figure 11:**
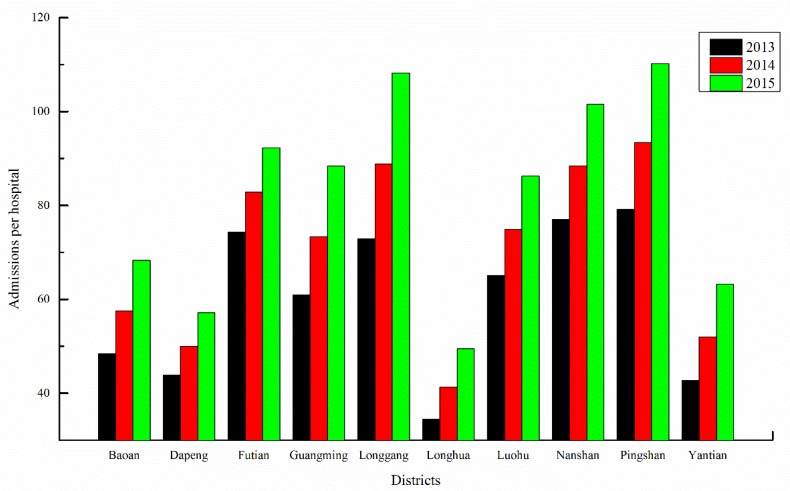
Mean admissions per hospital for each district in Shenzhen.

Figure 11 shows the IHD medical burden of each district from 2013 to 2015. Pingshan (79.2, 93.4, and 110.2) and Longgang (72.88, 88.83, and 108.25) are predicted to have the highest values of IHD medical burden among all of the districts, except in 2013, when Nanshan (77.04) and Futian (74.35) are predicted to have the second and third highest IHD medical burdens, respectively; Futian has the highest hospital admissions and IRs from 2013 to 2015. In other words, Pingshan and Longgang should have priority in the allocation of hospital resources and health-related financial investments in the near future.

## 4. Conclusions

This study aimed to understand the spatio-temporal patterns of IHD at the district level in Shenzhen from 2003 to 2012 using ArcGIS tools and SatScan software and to predict the IHD hospital admissions and incidence rate over the next three years using the GM (1,1) model to provide guidelines for the allocation of public health resources and the formulation of medical-related policies. The spatio-temporal analyses and predictions revealed interesting findings. We found that the IRs of each district exhibited a gradual increase from 2003 to 2012, which may have been caused by the rapid growth of the economy and/or the increasing traffic pollution. Furthermore, some neighboring areas exhibited similar temporal patterns. In the east of Shenzhen, the IRs from 2007 to 2009 were almost the same, and in the west of Shenzhen, outbreaks occurred during the same period in 2011. The west exhibited a variation with lower SRs, the east exhibited a variation with higher SRs, and the middle saw no changes in the level of SRs from 2003 to 2012. Futian, especially, always had the highest SR, and Longhua always had the lowest SR. However, each district exhibited its own pattern of change over the three-year intervals, although most of them had the same status from 2003 to 2012. We also observed that the hospital admissions for IHD showed distributions of spatio-temporal clusters that were not random. The primary clusters were Nanshan, Futian, and Luohu from 2008 to 2012. Although the hospital admissions for IHD maintained very similar MCs and SDEs from 2003 to 2012, they exhibited a slight northward movement, with the exceptions of 2003 and 2006, which was similar to the movement of the total population. From the combined analysis of the predictions of IHD hospital admissions and the current hospital distribution, Pingshan and Longgang are predicted to experience the most serious hospital service burdens with regard to IHD; Futian is likely to have the highest number of hospital admissions and the highest incidence rate related to IHD. These results can be used by urban public health officials and related decision makers to allocate public health resources and formulate prioritized medical-related policies.
